# An alpha7 nicotinic acetylcholine receptor agonist induces retinal neurogenesis and restores electrophysiological function following blast-induced ocular trauma in adult mice

**DOI:** 10.3389/fnins.2025.1710987

**Published:** 2025-11-26

**Authors:** Giovanna V. Nolasco de Carvalho, Jake B. Spitsbergen, David M. Linn, Cindy L. Linn

**Affiliations:** 1 Department of Biological Sciences, Western Michigan University, Kalamazoo, MI, United States; 2 Department of Biomedical Sciences, Grand Valley State University, Allendale, MI, United States

**Keywords:** neurogenesis, retina, electrophysiology, photoreceptor, retinal ganglion cell, blast injury, BrdU

## Abstract

**Introduction:**

Blast-induced ocular trauma is a major cause of vision loss in both civilian and military populations and effective treatments are limited by the adult mammalian retina’s inability to regenerate neurons lost to injury or aging. This study evaluated the neurogenic potential of PNU-282987, an alpha7 nicotinic acetylcholine receptor agonist, in an adult mouse model of blast-induced retinal trauma. We tested whether a delayed treatment paradigm, initiated after substantial neuronal loss occurred, could induce neurogenesis and restore retinal function.

**Methods:**

Adult mice received a single blast exposure, resulting in a significant reduction in cell counts across all retinal layers and decreased electroretinogram (ERG) amplitudes. One month after injury, when neuronal loss was fully established, daily eye drops of PNU- 282987 were administered for two weeks. Neurogenesis and cellular proliferation were assessed using BrdU incorporation and co-labeling with retinal ganglion cell and photoreceptor markers. Retinal function was evaluated with ERG recordings, and cell counts in treated retinas were compared with untreated blasted eyes and uninjured controls.

**Results:**

Delayed PNU-282987 treatment induced robust cellular proliferation and neurogenesis throughout the retina. Treated retinas showed significantly increased retinal ganglion cell and photoreceptor counts compared with untreated blast-injured eyes, reaching levels not significantly different from control retinas. Electrophysiological analysis revealed a significant recovery of ERG amplitudes, returning to baseline levels in most cases.

**Discussion/Conclusion:**

These findings demonstrate that PNU-282987-induced neurogenesis is sufficient to restore retinal structure and function even when treatment is initiated one month after blast injury. This work establishes a promising novel therapeutic approach for treating retinal trauma in adults.

## Introduction

With modern military conflict, blast-induced ocular trauma has emerged as a pervasive threat to vision across the globe (McMaster and Clare, 2021). A recent review found that blast injury is the most common method by which combat-related ocular trauma occurs, responsible for 64–84% of cases, with 51–69% of injuries associated with improvised explosive devices (IEDs) (Lee et al., 2023). Another study reported that combat ocular trauma resulting from blast exposure was highly prevalent, accounting for 80% of reported injuries (Gensheimer et al., 2021). Victims are not isolated only to military personnel, as ocular trauma is one of the leading causes of blindness worldwide (Bian et al., 2020; Rex et al., 2015). Ocular damage, which can occur as a result of blast trauma in the eye, yields consequences that can include vision loss which can contribute to a reduction in mobility, quality of life, mental health, and employment opportunities (Omar et al., 2022). There are four classifications of blast injury, including primary, secondary, tertiary and quaternary blast injuries. Primary and secondary traumas are the most prevalent. Primary blast injuries are induced via blast shockwaves from an explosion moving throughout the body, initiating pressure differentials across different tissues, and causing cell death, neuronal loss, and shearing (Rossi et al., 2012; Sosa et al., 2013). Primary blast injury is the focus of this study as it has been demonstrated to cause damage to the eye, optic tract, and retina, which has serious implications for vision loss (Hussain et al., 2021).

Sophisticated weaponry, such as missiles, grenades, and IEDs, can inflict a spectrum of severe optical injuries, including retinal hemorrhage, optic nerve disorders, and cell death (Jonak et al., 2025; Rana et al., 2023). The eye is uniquely susceptible to the implosion effects of the primary blast wave as it consists of fragile structures and vasculatures that are directly exposed to the environment (Zhang et al., 2023). This trauma initiates a progressive neurodegenerative response, which highlights a need for a therapeutic approach to protect or restore cells from damage. However, current treatments of blast-induced ocular trauma are fundamentally limited in their ability to restore lost visual function, as adult mammals do not have the capacity to regenerate neurons that have been lost as a result of disease, trauma or age (Silver and Miller, 2004; Benowitz and Yin, 2007). In some non-mammalian vertebrates however, activation of Müller Glia (MG) (glial cells that provide structural and homeostatic support to the neuronal areas of the retina) in response to retinal injury, can trigger neurogenesis to restore lost vision (Wan and Goldman, 2016). More specifically, in teleost fishes such as zebrafish, MG have been shown to undergo a process called interkinetic nuclear migration in response to injury, where MG in the immediate vicinity of the damage dedifferentiate, re-express retinal progenitor and stem cell markers, re-enter the cell cycle, migrate basal to apical, and divide once in an asymmetric division to generate a retinal progenitor. This newly produced progenitor cell will leave the cell cycle and is fated to differentiate into a mature retinal neuron (Lenkowski and Raymond, 2014). In chick embryos, regeneration occurs via progenitor cells that lie in the ciliary marginal zone at the edge of the retina as well as from reprogramming of MG (Doh et al., 2010). In Xenopus, regeneration has been shown to occur after activation of the MAPK signaling pathway through addition of fibroblast growth factor 2 (FGF-2) (Vergara and Del Rio-Tsonis, 2009). In adult mammals however, regeneration of new neurons across retinal layers in adult mammals does not typically occur and most treatments in adult mammals are focused on using a combination of pharmaceutical intervention and surgery to treat and reduce retinal degeneration (Maneu et al., 2022).

However, previous studies have demonstrated that the selective alpha7 nicotinic acetylcholine receptor (α7 nAChR) agonist, PNU-282987 (N-[(3’R)-1′-azabicyclo [2.2.2] oct-3′-yl]-4-chlorobenzamide hydrochloride), has induced neurogenesis in adult mammals (Webster et al., 2017, 2019; Stanchfield et al., 2020; Paris et al., 2021; Webster et al., 2021, 2022). PNU-282987 was originally developed for treatment of cognitive deficits associated with schizophrenia, specifically sensory gating (Hajós et al., 2004). However, further research showed that PNU-282987 caused cardiac regulatory issues in the human ether-a-go-go related gene (hERG), adversely affecting hERG potassium channels when applied systemically, rendering it impractical and unable to be used for human systemic delivery (Walker et al., 2006). In contrast, when PNU-282987 was applied as eye drops in adult rodents, it was found to increase photoreceptor and retinal ganglion cell populations in treated retinas when compared to untreated controls (Webster et al., 2017, 2019). Furthermore, PNU-282987 has been shown, in various retinal disease models, to replace photoreceptors associated with retinitis pigmentosa (RP) (Webster et al., 2021) and to replace lost RGCs associated with glaucoma (Paris et al., 2021; Webster et al., 2021; Luzadre et al., 2025). The neurogenic effect of PNU-282987 in adult rodents was attributed to dedifferentiation of MG cells (Webster et al., 2017, 2019; Stanchfield et al., 2020; Webster et al., 2022). However, based on immunocytochemistry studies and mRNA sequencing results in adult rodents, MG do not have α7 nAChRs (Stanchfield et al., 2020; Webster et al., 2022), prompting further investigation into the mechanism behind PNU-282987-based regeneration. As MG have apical processes that extend into the subretinal space and communicate with the RPE, and as α7 nAChRs are found on RPE cells (Maneu et al., 2010), it has been hypothesized that activated RPE secrete a signaling molecule(s) to induce the MG to dedifferentiate into progenitor cells that mature to replace lost neurons in the retina. This hypothesis has been supported by data that demonstrated conditioned media collected from cultured RPE cells previously treated with PNU-28297 is sufficient to induce neurogenesis in adult rodent retinas when it was injected into the vitreal chamber (Stanchfield et al., 2020; Webster et al., 2022).

Previous studies have also evaluated retinal physiological function shortly following blast-injury. The results from this study found that eye drop treatment with PNU-282987 was able to induce partial-recovery of retinal function after a single 35 psi blast to the eye (Spitsbergen et al., 2023) if PNU-282987 treatment began immediately after the blast. However, due to the design of this study, it was not clear if treatment results were neuroprotective or neurogenic. Previous studies that injected PNU-282987 into the vitreal chamber of adult rats demonstrated a neuroprotective effect on RGC survival when α7 nAChRs found on RGCs were directly activated (Iwamoto et al., 2013; Mata et al., 2015). This neuroprotective effect was initiated with PNU-282987 through activation of the PI3 kinase-Akt signaling pathway in RGCs (Iwamoto et al., 2013; Mata et al., 2015; Asomugha et al., 2010). The studies outlined in this current paper are designed to address this issue as treatment of the α7 nAChRs agonist did not begin until after significant blast-induced retinal cell loss had already occurred, thereby eliminating any potential neuroprotective effect.

This study used a combination of immunohistochemistry techniques and electroretinogram (ERG) recordings to address neurogenesis and functional activity in adult mice. One of the most common ERGs, the full field (flash) electroretinogram, sums up the electrical response of the entire retina with a flash of light to give an overall assessment of retinal function (Azarmina, 2013). The a-wave, known also as the receptor component of an electroretinogram, measures the activity of the photoreceptors. The b-wave measures bipolar cell activity, specifically the ON-type bipolar cells (Bhatt et al., 2023). Oscillatory potentials are high-frequency, low-amplitude responses found on the rising phase of the b-wave and can be used to identify changes in inner retinal cell activity as well as circulation of the retina and retinal pathologies (Bellini et al., 1995; Liao et al., 2023). Another type of ERG measurement lies in the Flicker Frequency Series (FFS), which can allow for insight into changes in light sensitivity and perception (Park et al., 2023). More specialized types of ERGs, including the photopic negative response (PhNR) and pattern electroretinogram (pERG), are equipped to assess visual function in different components of the eye. The light-adapted PhNR, consisting of a negative wave following the a- and b-waves, serves to assess RGC function as well as the parvocellular color pathway, giving an insight to study the impact of neuropathy and subsequent treatment on the innermost retinal layer (Ventura and Porciatti, 2006; Prencipe et al., 2020). The pERG amplitude is measured by the difference between P1 and N2 markers on the first positive and second negative deflection of the pERG waveform, which evaluate the health of RGCs. More specifically, the P1 component details pre-ganglionic bipolar cell and amacrine cell activity, whereas the N2 component directly examined RGC functionality (Marmoy and Viswanathan, 2021). Finally, electroretinograms can also provide information on latency (implicit time) – the time between the onset of stimuli and subsequent response. Latency measurements are an important clinical tool that allows researchers further insight into ocular health and neurological deficits, such as in cases of retinal disease (Berson, 1981; Fortune et al., 1999; Huang et al., 2023). More specifically, delays found in latency or implicit time can be an early indicator of neural dysfunction, which can inform clinicians about potential treatments for various diseases and disorders, such as glaucoma (Parisi, 2001). Evidence of blast-induced trauma impacting latency has been reported (Mohan et al., 2013; Vest et al., 2019). However, as primary blast injury is usually comorbid with other classifications of blast injury (Kluger, 2003; Housden, 2012), the effect of primary blast injury on visual latency itself is still poorly understood.

In this current study, treatment with PNU-282987 did not begin until after blast damage to the retina had already occurred. Previous studies have demonstrated significant loss of retinal cells in all cellular layers and significant decreases in ERG waveform recordings 1–4 weeks after a single 35 psi pressure blast is delivered to adult mouse eyes from a modified paintball gun (Spitsbergen et al., 2023). Treatment with PNU-282987 did not begin in this current study until 1 month after the initial blast exposure was delivered, ensuring that significant loss of retinal cells had already occurred (Spitsbergen et al., 2023). Using this design, we strictly evaluate the neurogenic potential of PNU-282987 treatment following blast injury for the first time. The objective of this study is to provide support that administration of PNU-282987 following blast-induced ocular trauma in adult mice induces retinal neurogenesis and leads to a significant anatomical restoration of electrophysiological function in the adult mammalian retina.

## Materials and methods

### Animals

Wild-type 129/SVJ male and female mice were used in this experiment, purchased from Jackson Laboratories (Bar Habor, ME, United States). The subjects were aged between 3–6 months and weighed 20–25 grams. Mice were bred and kept in Western Michigan University’s animal facility and given food and water ad libitum in a 12-h light/dark cycled environment. Mice were cared for in accordance with the standards set by the Institutional Animal Care and Use Committee (IACUC) at Western Michigan University. All experiments involving animal subjects were also conducted according to the National Institute of Health (NIH) Guide for the Care and Use of Laboratory Animals (NIH Publications No. 80–23) revised in 1996. Four different cohorts of mice were used for this study, which included control untreated animals, control animals that only received PNU-282987 treatment, experimental animals exposed to blast trauma, and experimental animals that received blast trauma followed by PNU-282987 treatment. All control untreated animals came from wild type adult animals that had no treatment or blast exposure to either eye. Likewise, all PNU-282987 only treatments were delivered to both eyes of a wild type adult mouse that had no blast exposure to either eye.

### Blast injury model and procedure

**Figure 1:**
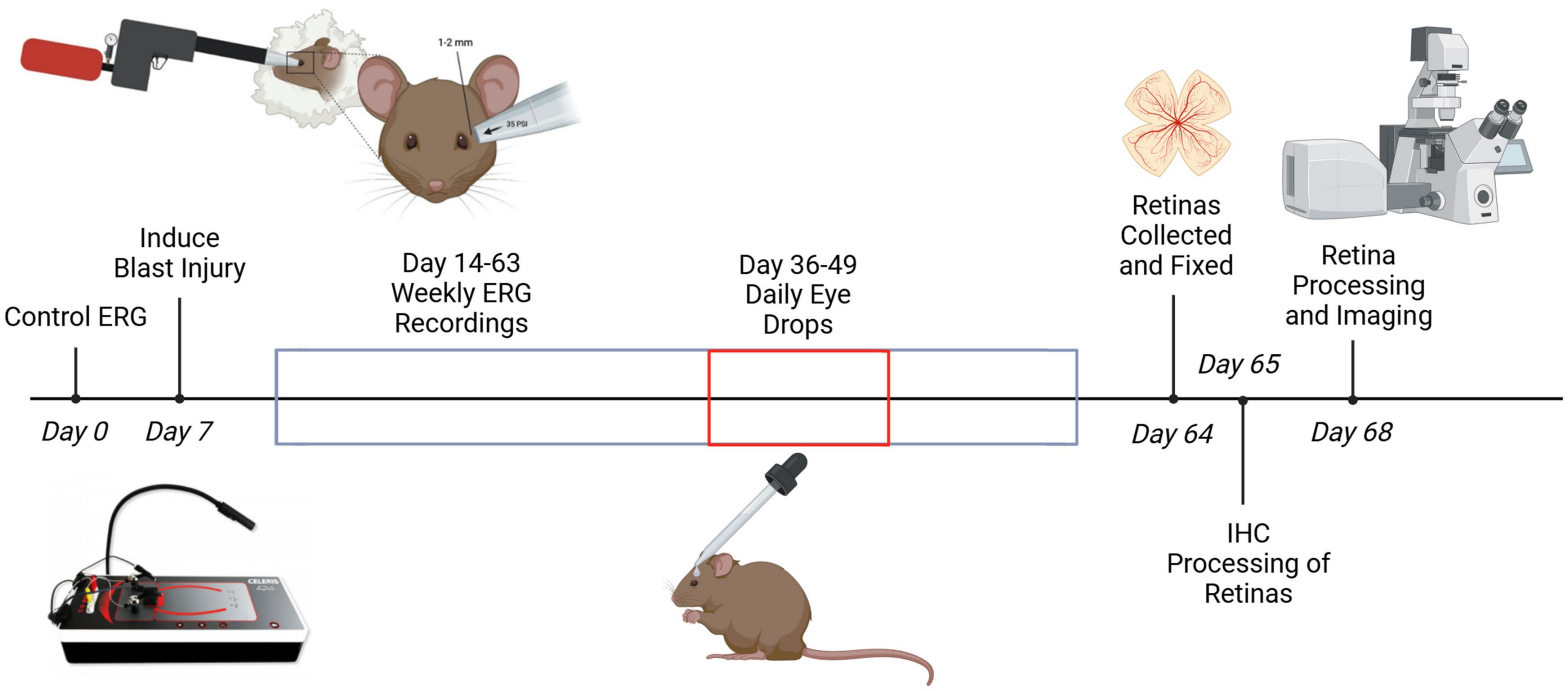
A timetable illustrating when blast exposure was implemented, eye drop application conducted, and ERG recordings obtained. At the end of each experimental treatment, animals were sacrificed, and retinas were collected and processed for immunohistochemistry. A schematic of how the modified paintball gun was used is shown above the timeline. *Created with BioRender.

To mimic damage of a landmine explosion blast wave, a commercially available paintball gun (Invert Mini, Empire Paintball, NJ, USA) was modified according to Hines-Beard et al. (2012), and consistent with the modified paintball gun used by Spitsbergen et al. (2023). The barrel was machined to be shorter, with the front of the barrel modified to focus the compressed air blasted to the size of a mouse eye. Prior to blast experimentation, animals were anesthetized using ketamine/acepromazine/xylazine (KAX) at a concentration of 66/1.3/6.6 mg/kg body weight through an intraperitoneal injection. After approximately 10 min, the lack of a toe pinch response confirmed that animals were anesthetized completely. They were placed in an open mouse holder, heavily cushioned with cotton and the head of each mouse was restrained to ensure proper alignment of the eye (Spitsbergen et al., 2023), avoid brain damage, avoid whiplash, and any other adverse effects sustained from blast pressure. The barrel of the gun was placed approximately 1–2 mm from the eye of the mouse, directly over the center axis of the cornea, aimed at a 90-degree angle directly down the animal’s line of sight, and one single blast of 35 PSI pressurized air was delivered to the left eye. The right eye was not exposed to blast injury and served as an internal control for electrophysiological analyses. However, untreated control retinas were obtained from a separate cohort of mice that did not receive a blast nor PNU-282987 treatment. Mice were provided with 35 mg/mL acetaminophen in their drinking water for 7 days following blast injury. A timeline representative of experimental procedures is included as Figure 1.

### Eye drop treatment and retina preparation

One month after blast-injury induction, eye drops in PBS containing 1% dimethyl sulfoxide (DMSO) and 1 mM PNU-282987 were applied once bilaterally daily for 2 weeks, with approximately one drop of solution administered *in vivo* onto the external surface of the eye using a 1 mL plastic pipette. Control group animals received PBS eye drops containing 1% DMSO. Eye drops for control and experimental groups also included BrdU at a concentration of 1 mg/mL BrdU to label mitotically active cells (Linn et al., 2018). All mice were treated at the same time each day in dark-adapted conditions. This was conducted according to the timeline outlined in Figure 1. Previous dose response studies using several concentrations of PNU-282987 have been conducted (Webster et al., 2017). In dose response studies, concentration of PNU-282987 between 0 and 2 mM was applied as daily eye drops and the percent of BrdU positive cells in the retina resulting from these treatments were calculated. Eye drops containing 1 mM PNU-282987 elicited the maximal effect. To ensure that eye drops containing PNU-282987 reached the retina, previous HPLC MS/MS studies demonstrated that eye drop delivery reached the retina at detectable levels (Mata et al., 2015). In separate studies, other routes of administration were analyzed. For instance, previous studies injected PNU-282987 into the vitreal chamber to observe effects in the retina. However, when directly injected into the vitreal chamber in adult rodents, PNU-282987 comes in direct contact with RGCs which contain α7 nAChRs and leads to a neuroprotective effect of RGCs (Iwamoto et al., 2014), separate from the neurogenic effect induced by PNU-282987 when applied as eye drops. Control untreated animals received PBS eye drops containing 1% DMSO. Following the conclusion of eye drop treatment, animals were euthanized via CO_2_ asphyxiation 2 weeks later. Afterwards, the eyeballs were removed, retinas were excised, flat-mounted onto Sylgard dishes using cactus needles and fixed overnight in 4% paraformaldehyde (PFA) at 4 degrees Celsius for immunohistochemistry processing.

### Immunohistochemical analysis

As previously described (Webster et al., 2017, 2019, 2021), antibody labeling was performed on fixed retinal tissue. Antibody staining was conducted for immunohistochemical (IHC) labeling using sheep anti-bromodeoxyuridine (BrdU) (Abcam 1893) to label mitotically active cells; rabbit polyclonal anti-Recoverin (Invitrogen PA5-110287) to label photoreceptors; and rat anti-Thy 1.2 (BD Biosciences A553000) to label mouse retinal ganglion cells (Table 1). Retinal tissue was labeled with primary antibodies in PBS containing blocking serum overnight at 4 °C. Retinas were then rinsed with PBS and incubated with secondary antibodies using Alexa Fluor 488 or 594 antibodies at room temperature for 2 h. The specific secondary antibodies used, and their corresponding dilutions, are listed in Table 1. Post-IHC, retinas were either sectioned into 40-micron strips and positioned so that all 3 nuclear retinal cell layers faced upwards or were flat-mounted onto glass slides with the RGC layer facing up. For cell counting, the fixed retinas were stained with 4′,6-diamidine-2′-phenylindole dihydrochloride (DAPI; Sigma-Aldrich). Retinas were suspended in 50% PBS and 50% glycerol on a glass coverslip for imaging on a confocal microscope.

**Table 1:** A list of primary and fluorescent secondary antibodies used for staining of retinal sections and flat mounts.

Antibodies	Source	Identifier	Dilution
Sheep anti BrdU	Abcam	Ab1893	1:125
Rabbit polyclonal anti Recoverin	Invitrogen	PA5-110287	1:100
Rat anti Thy 1.2	BD Biosciences	A553000	1:200
Alexa Fluor Conjugated Secondary	Invitrogen	A21208; A21207; A11015; A11016	1:300

### Microscopy and cell counts

A Nikon C2+ scanning laser confocal microscope (Tokyo, Japan) was used to visualize the fixed and immunostained retinal tissue. DAPI-stained cells were counted from 5 different quadrants of each sectioned retina 4 mm from the optic nerve head. An average cell count was obtained from 200-μm^2^ confocal images (Iwamoto et al., 2014; Mata et al., 2015; Webster et al., 2021). The average number of cells in the outer nuclear layer (ONL), inner nuclear layer (INL), and ganglion cell layer (GCL) from retinas exposed to blast was compared to experimental PNU-282987 treated retinas after blast injury and to control undamaged retinas.

### Electroretinogram recordings

Flash electroretinograms (ERG), Flicker Frequency Series (FFS), Photopic Negative Response (PhNR), and Pattern Electroretinograms (pERG) were recorded in accordance with the timeline represented in Figure 1. Animals were anesthetized with KAX prior to any recordings. Ten minutes after KAX delivery, the animal’s behavior response was observed, and a lack of response to a toe pinch indicated that animals were anesthetized completely. Once anesthetized, mice were placed on the Celeris Diagnosys Rodent ERG system (Diagnosys, LLC, MA, United States) for recordings. The built-in heating elements in the system were used to maintain the anesthetized animal’s body temperature at 37 degrees Celsius. The pupil reflex was anesthetized with proparacaine to prevent blinking and dilated with 1% tropicamide ophthalmic solution (Bausch and Lomb, NY, United States). For scotopic recordings, animals were dark-adapted for 8–10 h overnight prior to recording. To decrease recording impedance and to prevent the animal’s eyes from drying out, a drop of 0.3% Hypromellose gel (Alcon Laboratories Inc., TX, United States) was placed on the contact surface of each stimulating/recording electrode, which was then placed subsequently on the cornea of each eye. Grounding electrodes were placed at the base of the tail and in the tongue. Responses were recorded from both eyes simultaneously in the dark for flash ERGs, FFS, and pERG recordings. Recordings of dark-adapted mice were performed under red LED illumination as mice cannot detect this wavelength of light (Peirson et al., 2018). The flash intensity of the light delivered for dark-adapted ERG recordings was 1.0 cd/m^2^, and 3 responses were averaged for each eye to represent an “N” of 1. Dark-adapted FFS recordings involved 3 separate recording steps of high frequency flash stimulation including frequencies of 5, 15, and 20 Hz. For each step, the intensity of the flash was 3.0 cd/m^2^, and 50 responses were averaged for each eye representing an “N” of 1. Dark-adapted pERG recordings used a checkerboard contrast pattern stimulus. Six hundred responses were obtained and averaged for each “N.” The stimulus intensity for the pERG recordings was 50 cd/m^2^. PhNR recordings were taken in light-adapted conditions. These recordings used a prolonged green flash intensity of 40 cd/m^2^ and a flash of 20 cd/m^2^ white light, where 100 responses were averaged for each eye representing an “N” of 1.

The ERG responses were analyzed using Espion V6 software (Diagnosys, LLC, MA, United States) from each animal before blast, and for each week following blast for 8 weeks. For dark-adapted flash ERGs, the a-wave amplitudes were measured from baseline to the trough of the a-wave. B-wave amplitudes were measured from the trough of the a-wave to the peak of the b-wave. Oscillatory potentials (OPs) were isolated by the Celeris Espion V6 software with a bandpass filter of 75–300 Hz. The amplitudes of OP1, 2 and 3 were measured from the preceding trough to the peak. FFS recording amplitudes were measured from the base to the peak response. For light adapted PhNRs, amplitudes were measured from the baseline to the trough immediately following the b-wave. In pERG recordings, amplitudes were measured from the peak of the P1 amplitude to the trough of the N2 amplitude. Latency measurements were also assessed for insight into cellular function. Changes in latency often occur prior to structural damage, thus, they potentially allow for early diagnosis of damage to the neural retina (Bach et al., 2012). Latencies of pERG recordings were measured from the onset of stimulation to the peak of the P1 for P1 latency, as well as to the trough of the N2 response for N2 latency.

### Statistical analysis

One-way ANOVA was used for multiple comparisons to statistically measure cellular loss with Tukey’s *post hoc* tests using statistical software (GraphPad Software Inc., San Diego, CA). For normalized data, statistical analysis was performed using Kruskal–Wallis nonparametric analysis of variance with post hoc comparisons (Dunn’s test). All error bars represent SEM and exact *p*-values are listed for each statistical comparison. Bar graphs were plotted with statistical software (GraphPad Software, Inc.). Sample sizes were determined using an *a priori* power analysis (GPower 3.1). Based on calculations using a two-tailed hypothesis test, a minimum of 6 animals per group was required to achieve 80% power at alpha = 0.05. As preliminary data demonstrated no significant difference between male and females when analyzing cell morphology or ERG recordings after blast exposure or after blast exposure with PNU-282987 treatment, a total of 6–8 (equal numbers of males and females) were used for each condition analyzed in this study and the data was pooled together. *p < 0.05 was statistically significant.

## Results

### Effect of blast exposure and PNU-282987 on neural layers of the retina

**Figure 2:**
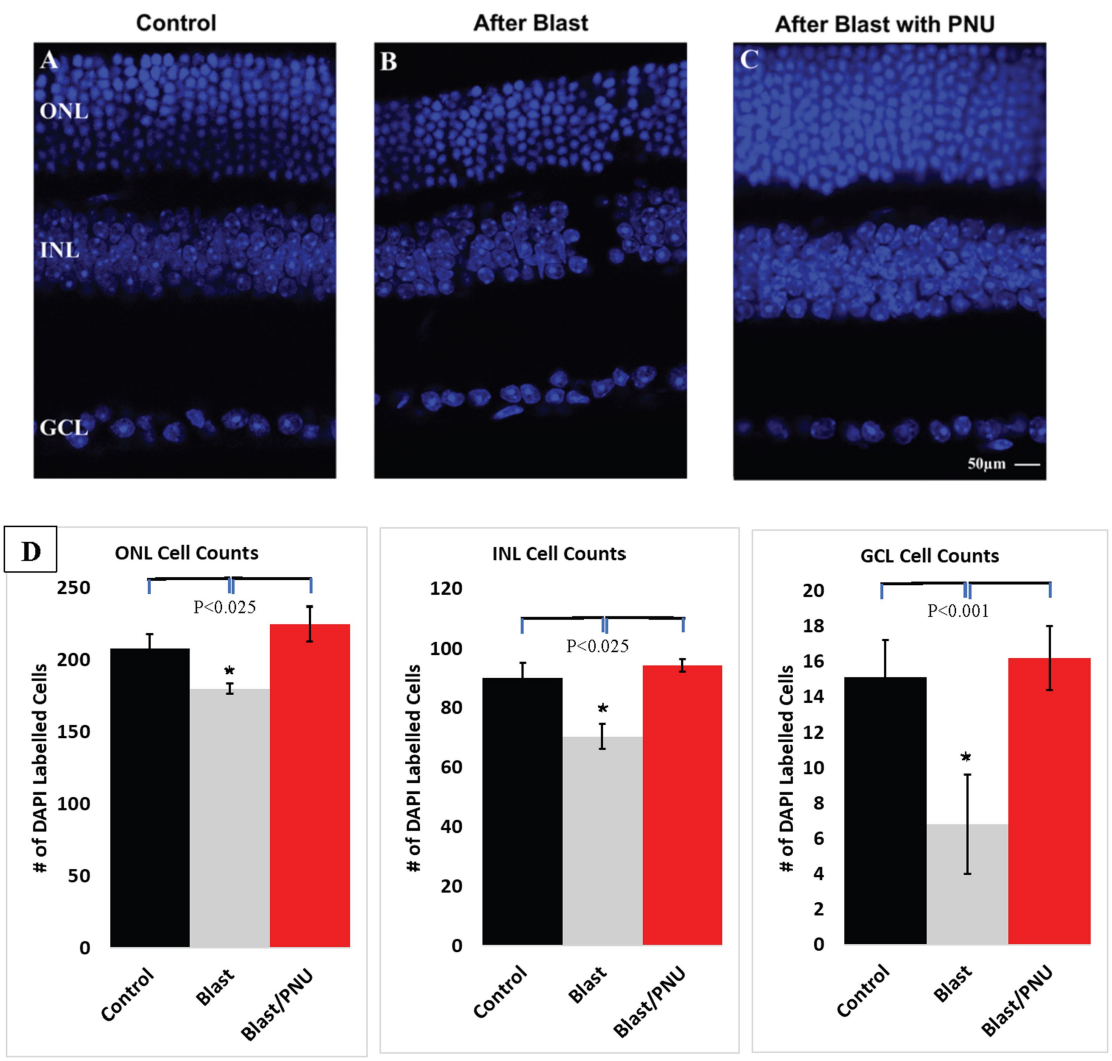
Effect of Blast Exposure in retinal sections of the ONL, INL, and GCL in all three retinal layers. Cells were labeled DAPI (blue) to visualize cell bodies (top images). Panel **(A)** shows an untreated control retina. Panel **(B)** represents an untreated, blasted retina. Panel **(C)** represents a blasted retina after 2 weeks of PNU-282987 treatment that did not begin until 1 month after blast exposure. Quantification of cell counts obtained from each nuclear layer is summarized below **(D)**. Error bars represent SEM. * represents significant difference from control and from blast exposure followed by PNU-282987 treatment. *N* = 8 for each condition. Scale bar represents 50 μm.

Consistent with the findings of Spitsbergen et al. (2023), after a single 35 PSI blast was delivered to an adult mouse eye, cell counts significantly decreased in all layers within 2 weeks. However, if eyes were treated with PNU-282987 eye drops, which did not begin until after significant blast-induced cell death had occurred, cell counts returned to levels that were not significantly different from untreated controls. In Figure 2, DAPI stained cells were counted. ONL cell counts under control conditions averaged 208.15 cells ± 10.2 from 200-μm^2^ confocal images. The INL cell counts averaged 90.1 ± 5.2, and the GCL counts averaged 15.1 ± 2.1 from confocal images. After blast damage occurred, ONL cell counts significantly decreased from control counts to an average of 180.1 ± 3.4, INL cell counts decreased to an average of 70.4 ± 4.1, and GCL cell counts decreased to an average of 6.8 ± 2.8. Following blast exposure and PNU-282987 treatment, ONL cell counts significantly increased to an average of 225.22 ± 12 cells, INL cell counts increased to an average of 94.4 ± 2.1, and GCL cell counts increased to an average of 16.2 ± 1.8. Following blast and PNU-282987 treatment, all cell counts returned to values comparable to those of control untreated and uninjured retinas. In sections treated only with PNU-282987, the average cell count in the ONL increased by an average of 18.2% ± 2.4, the average cell count in the INL increased by an average of 25.2% ± 6.8 and the average cell count in the GCL increased by an average of 26.4% ± 5.1 as previously reported (Webster et al., 2017, 2019).

**Figure 3:**
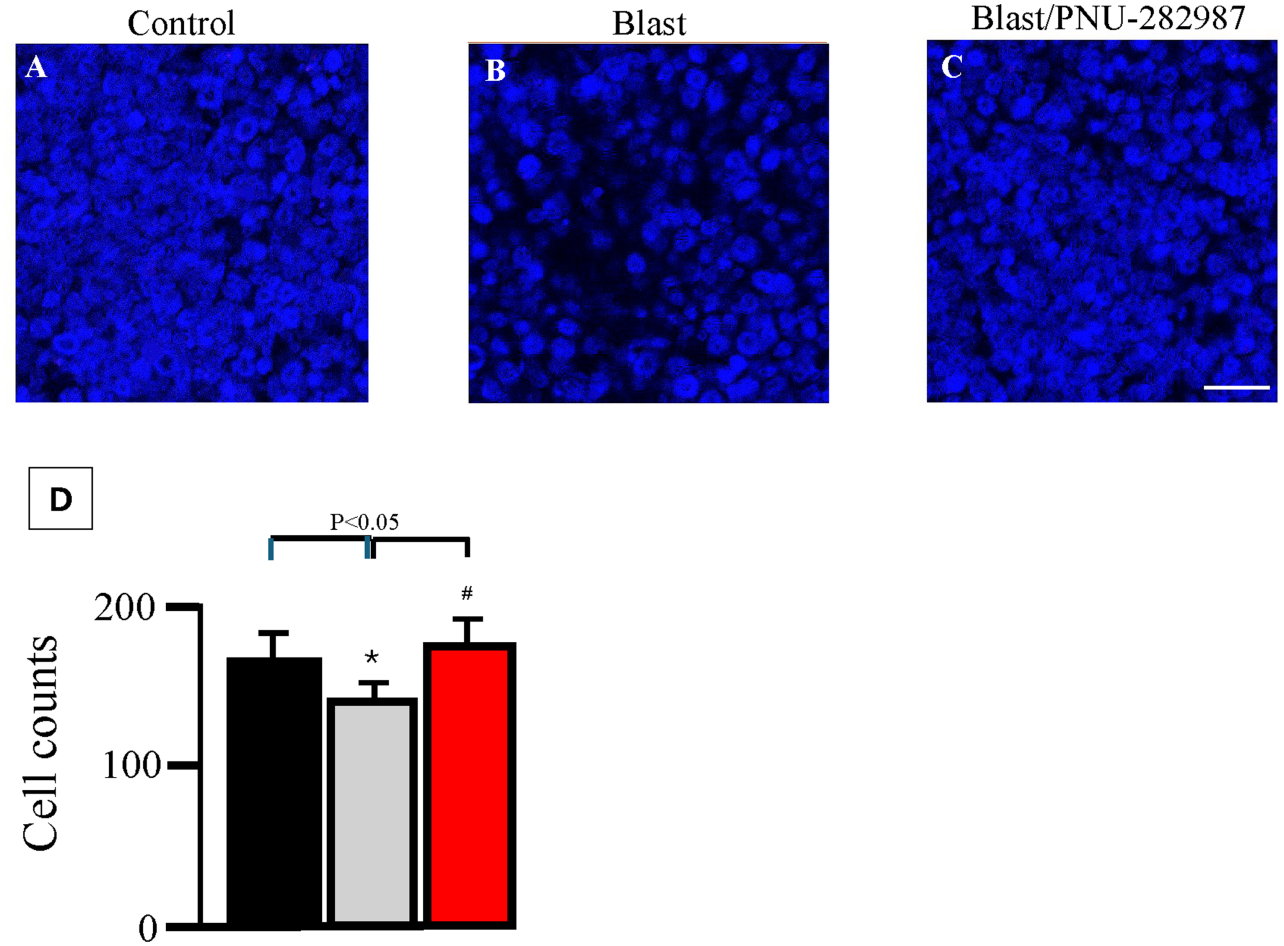
Effect of blast exposure in flat mounts of the GCL. Cells were labeled DAPI (blue) to visualize cell bodies. Panel **(A)** shows an untreated control retina. Panel **(B)** represents an untreated, blasted retina. Panel **(C)** represents a blasted retina after 2 weeks of PNU-282987 treatment, applied after neuronal loss from blast damage had already occurred. Quantification of average cell counts is illustrated in **(D)** (black = control untreated, gray = counts after blast exposure, red = average counts obtained after blast exposure following PNU-282987 treatment). Error bars indicate SEM. * represents significance from untreated control conditions. # represents significance from blast conditions. *N* = 8 for each condition. Scale bar represents 50 μm.

Flat mounted retinas were also evaluated under each condition to look at RGCs. In Figure 3A, DAPI stained cells were counted in flat mounted retinas obtained under control untreated conditions (Figure 3A), after blast exposure (Figure 3B), and after blast exposure following PNU-282987 treatment (Figure 3C). Quantification of these conditions was summarized in Figure 3D. GCL cell counts under control conditions averaged 180 ± 5.9 from confocal images. The GCL cell counts after blast damaged occurred significantly decreased from control to an average of 144.8 ± 3.9. Once PNU-282987 treatment was applied after blast exposure, cell counts in the GCL increased to an average of 184.2 ± 4.7. The blast only group cell counts were significantly different from untreated controls, but there was no significant difference between control and blasted tissue that was followed by delayed PNU-282987 treatment. In flat mounted tissue only treated with PNU-282987, the average cell count was 198 ± 2.9, representing a significant increase from control untreated conditions as previously reported (Webster et al., 2017, 2019).

### PNU-282987-induced mitotic activity in neural layers of the retina

**Figure 4:**
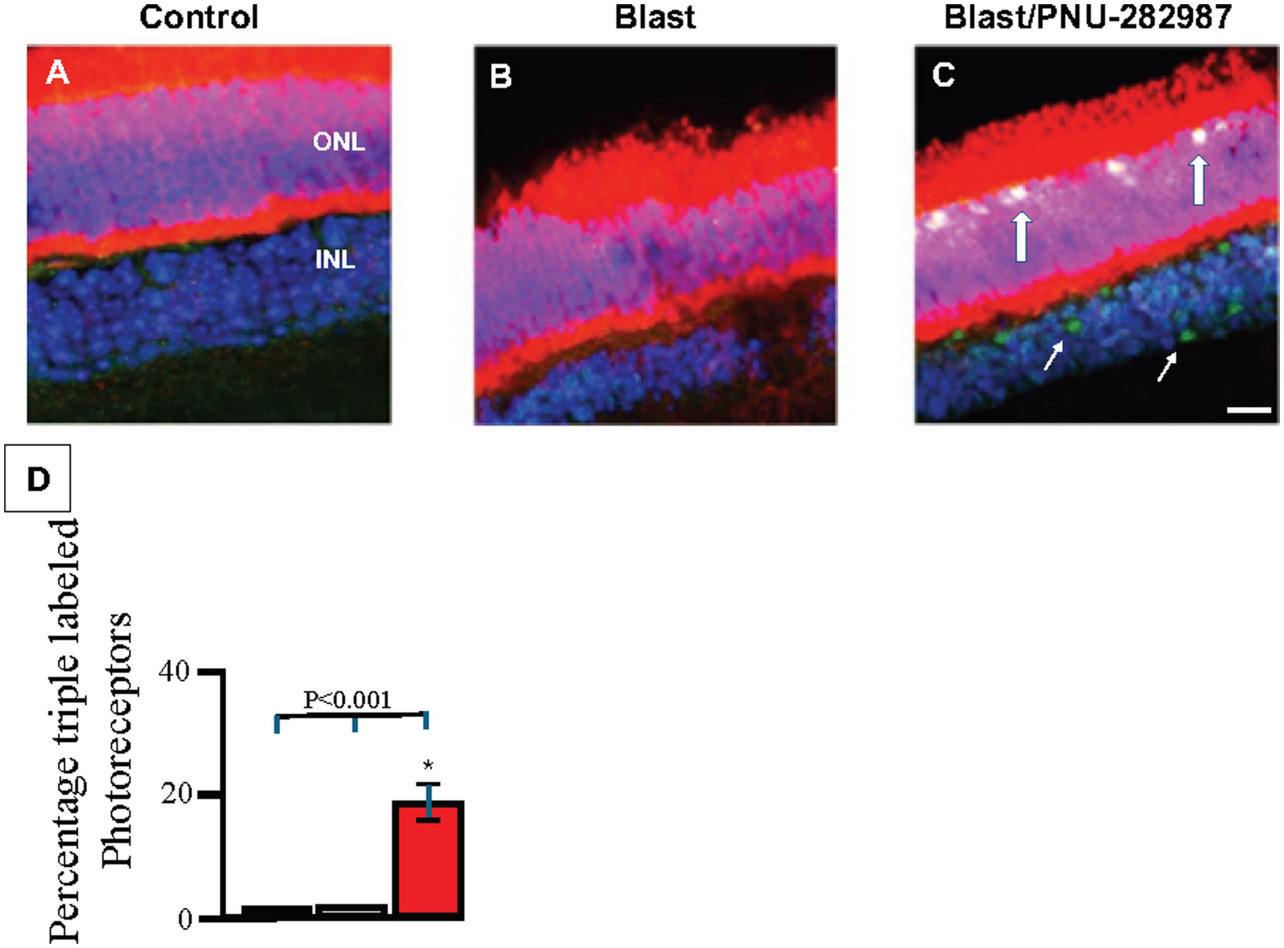
Neurogenesis in the ONL and INL of retinal sections. Retinas were processed using IHC with primary antibodies against recoverin (red) and BrdU (green) and then counterstained with DAPI (blue). Panel **(A)** shows a control retina on Day 60. Panel **(B)** shows an untreated, blasted retina on Day 60. Panel **(C)** shows the effect of PNU-282987 on mice that received blast-induced ocular trauma at Day 60. Thick, white arrows show triple labeling of photoreceptors with recoverin, BrdU, and DAPI. Thin, white arrow show BrdU localized in the INL. Panel **(D)** summarizes the percent of double labeled photoreceptors measured in the ONL that resulted from PNU-282987 treatment after blast exposure. The black bar represents control untreated retinas, gray represents results after blast exposure and red indicates the percent of triple labeled photoreceptors after blast followed by treatment with PNU-282987. Each bar average was obtained from 8 animals. Error bars = SEM. * represents significant difference from control and from blast exposed condition. Scale bar represents 30 μm.

**Figure 5:**
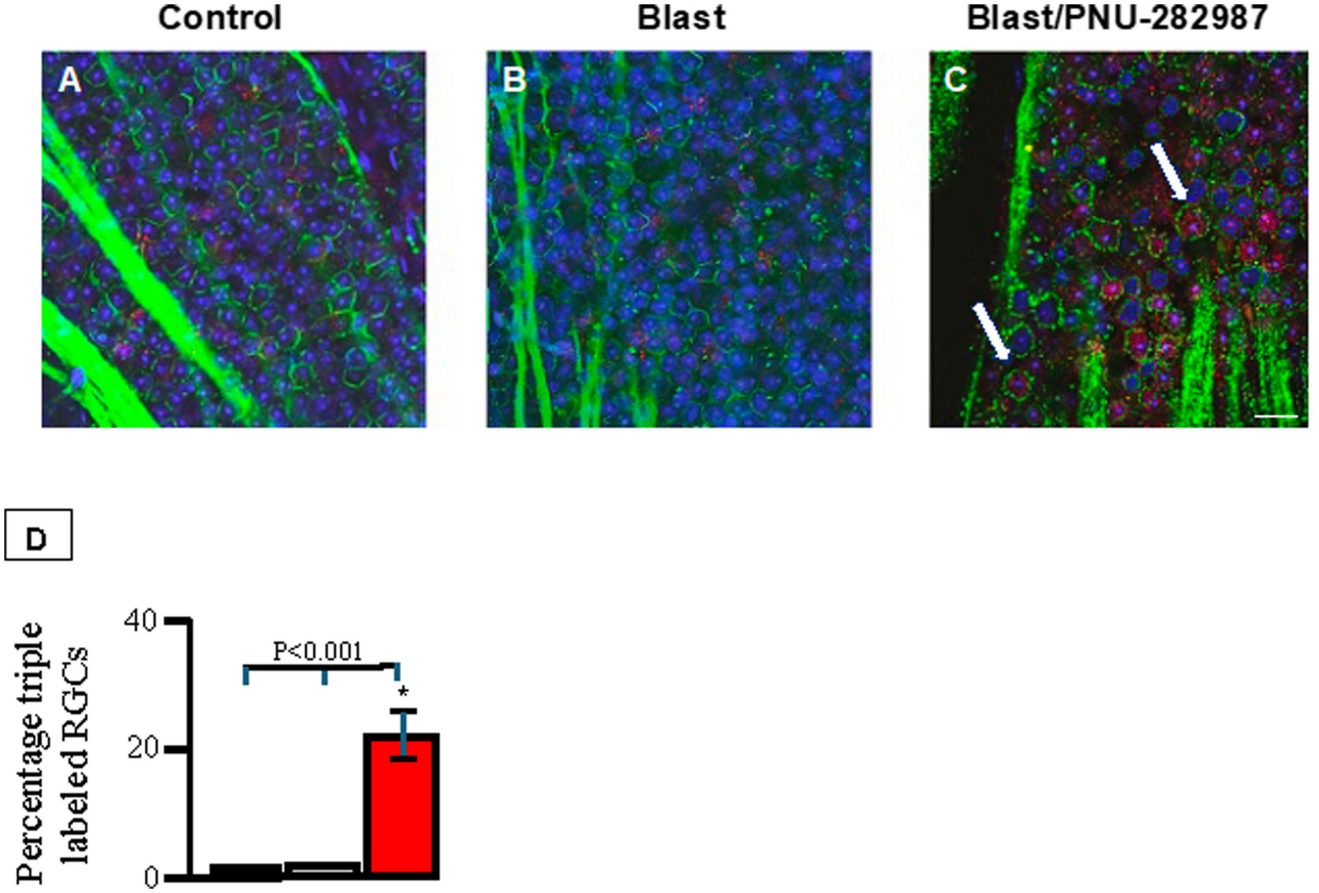
Neurogenesis in the GCL of flat mounted retinas. Retinas were processed using IHC with primary antibodies against BrdU (red) and Thy 1.2 (green). Retinas were counterstained with DAPI (blue). Panel **(A)** shows a control retina. Panel **(B)** shows a blasted retina with no PNU-282987 treatment. Panel **(C)** shows the effect of PNU-282987 on mice that received blast-induced ocular. Thick, white arrows represent triple labeled RGCs with Thy 1.2, BrdU, and DAPI. Panel **(D)** summarizes the percent of triple labeled RGCs measured in the GCL that resulted from PNU-282987 treatment after blast exposure. The black bar represents the average obtained from control untreated retinas; gray represents results after blast exposure and red indicates the percent of triple labeled RGCs after blast followed by treatment with PNU-282987. Each bar average represents an average obtained from 8 animals. * represents significant difference from control conditions and from blast exposed condition. Error bar = SEM. Scale bar represents 30 μm.

Compared to untreated control retinas, retinas with a delayed treatment of PNU-282987 following blast injury showed a significant increase in mitotically active cells and evidence of new retinal neurons. These findings are consistent with previous work demonstrating that PNU-282987 eye drops can induce neurogenesis across all retinal layers in healthy retinas (Webster et al., 2017, 2019, 2021). More specifically, BrdU incorporation was seen in all retinal cell layers after PNU-282987 treatment following blast trauma (Figures 4, 5). In retinal sections (Figure 4), retinas were counterstained with DAPI (blue) and labeled with antibodies against recoverin (red) to label photoreceptors and against BrdU (green) to label mitotic activity. After blast induced ocular trauma, cell counts significantly decreased by an average of 18.9% ± 2.6 compared to controls. In control retinas, as well as blasted retinas not receiving treatment, there was no sign of photoreceptors co-labeled with BrdU. However, following induction of blast injury and treatment with PNU-282987, cell counts increased significantly back to control levels in the ONL and INL (Figure 4). In addition, 15.8% ± 2.5% of total photoreceptors in the ONL were co-labeled with BrdU and recoverin (Figure 4C), indicating that BrdU positive photoreceptors were generated following treatment with PNU-282987. There was no evidence of co-labeled photoreceptors under control untreated conditions or after blast exposure (Figures 4A,B). The percent of triple labeled photoreceptors under control conditions, after blast exposure and after blast with PNU-282987 treatment is summarized in Figure 4D. In retinas that were only treated with PNU-282987, 18.5% ± 8.2 of photoreceptors co-labeled with BrdU and recoverin as previously reported (Webster et al., 2017, 2019).

PNU-282987 application also induced BrdU positive RGCs (Figure 5). Flat mounted retinas were immunostained with antibodies against Thy 1.2 to label RGCs, BrdU to label mitotic activity and counterstained with DAPI. In flat mounted retinas, following blast injury, retinal cell counts decreased significantly by 23.2 ± 4.5 relative to controls (100%) within the RGC layer. In contrast, following administration of PNU-282987, cell counts significantly increased to 115% ± 7.2 compared to controls. In addition, 28.2% ± 3.6 of RGCs were BrdU positive after blast injury followed by PNU-282987 treatment, indicating neurogenic activity (Figure 5). In flat mounted retinas that were only treated with PNU-282987, 26.5% ± 8.2 of RGCs co-labeled with BrdU and Thy 1.2 as previously reported (Webster et al., 2017, 2019).

### Functional recovery evaluated by ERG recordings: dark adapted flash

**Figure 6:**
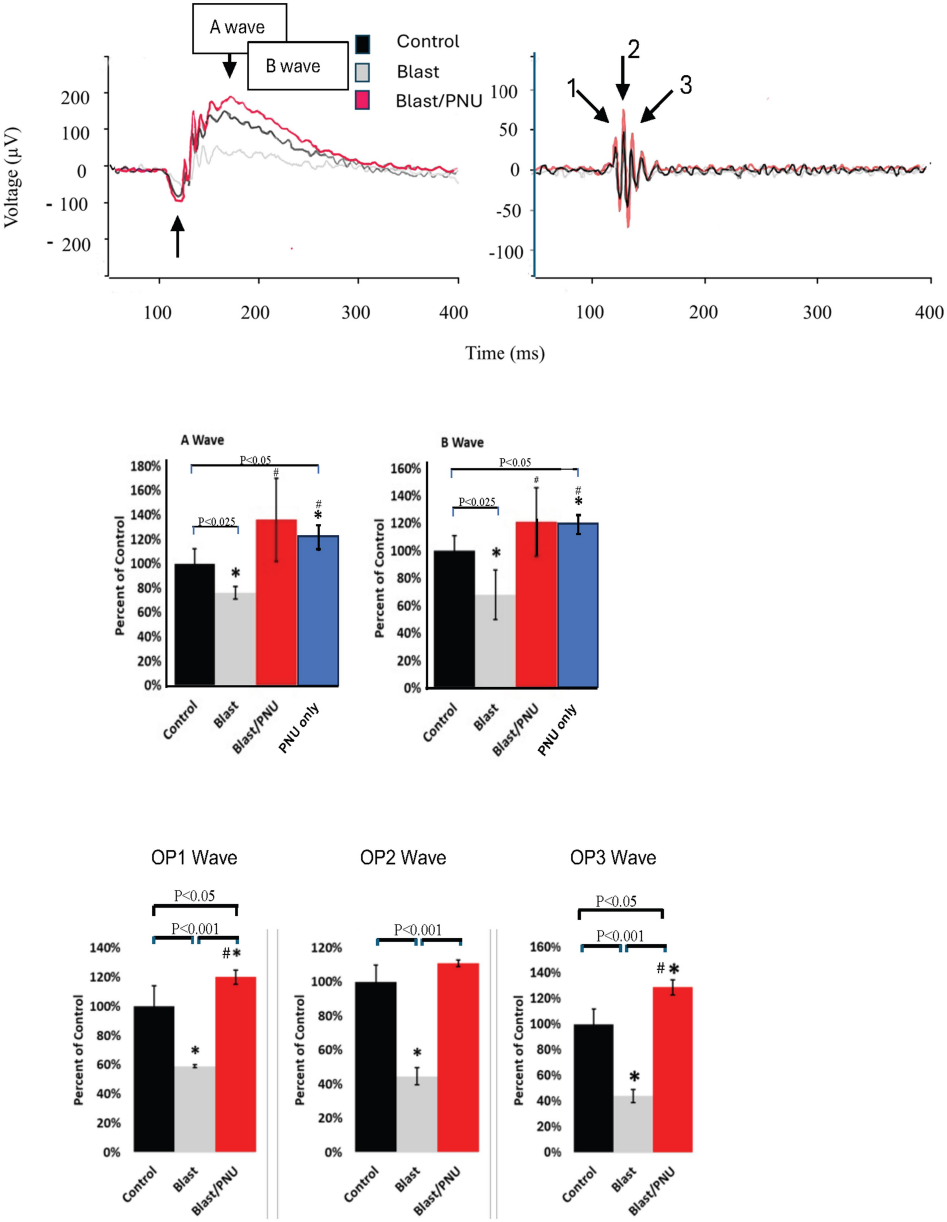
Recovery of ERG amplitudes and oscillatory potentials after treatment with PNU-282987 in blast exposed mice. The ERG traces demonstrate the effects of blast exposure and PNU-282987 treatment. The superimposed ERG traces were obtained from a single animal and contain traces from control (black), blast only (gray), and blast with PNU-282987 (red) treated mice. Quantification of average traces shown below (*N* = 6–8). In summarized bar graphs for the A- and B-wave, the blue bar represents a cohort of animals only treated with PNU-282987. * represents significant difference from control. # represents significant difference from the blast effect. Error bar = SEM.

Visual function of the retina was assessed using electrophysiological analysis via the Celeris Diagnosys System. The ERG waveforms generated from a single flash of 1.0 cds/m^2^ in a dark-adapted animal are shown in Figure 6. In the top left images of Figure 6, the three superimposed average ERG traces and represent the ERG’s a- and b-waves as well as the oscillatory potentials that are visible on the rising phase of the b-wave. The top right panel in Figure 6 shows the OPs 1,2 and 3, isolated via a 75–300 Hz bandpass filter from waves shown on the top left. The black waveform represents the baseline recording for the animal prior to blast injury. The gray waveform was recorded 1 week after blast exposure to the eye. The red wave is a recording taken 8 weeks post-blast injury. PNU-282987 drop treatment did not begin until 4 weeks after the blast injury to ensure significant cell loss had already occurred before PNU-282987 treatment began. All waveform recordings were obtained from the same adult mouse, while the bar graphs represent mean results from each treatment population (Figure 6). As demonstrated by these ERG recordings, the induced blast caused a significant decrease in all ERG waveforms examined. However, this decrease was eliminated after treatment with PNU-282987.

A quantification of these effects is illustrated in the bar graphs of Figure 6. ERG waveform analysis revealed that the a-wave amplitude significantly decreased by an average of 24% ± 5%, the b-wave amplitude decreased by an average of 32% ± 18%, and oscillatory potentials (1, 2, and 3,) decreased by an average of 41% ± 1, 55.4% ± 5, and 55.8% ± 5%, respectively, following blast injury. However, amplitudes recovered to levels significantly similar to control levels when the blast was followed with PNU-282987 treatment. The a-wave amplitude had an average recovery of 136% ± 34% compared to control and the b-wave amplitude recovered to 121% ± 25% in comparison to control. OP1, 2, and 3 recovered to 120% ± 5, 111% ± 2, and 129% ± 6% when compared to uninjured control recordings. The values of OP1 and OP3 after blast and PNU treatment were statistically greater than control untreated values, as demonstrated by the quantification summarized in Figure 6, supporting the hypothesis that PNU-282987 treatment can affect visual processing to increase responsiveness to light stimuli. In an additional study, ERG recordings were obtained from a cohort of animals that were only treated with PNU-282987. These results are summarized for the a- and b-wave in the blue bar graph shown in Figure 6. PNU-282987 treatment by itself, significantly increased the amplitudes of the a- and b-wave corresponding with the increase of photoreceptors reported in Figure 4. No significant change in latencies was found between control, blast-only, or blast with PNU-282987 treatment conditions in flash ERG recordings.

### Functional recovery evaluated by ERG recordings: flicker frequency series

**Figure 7:**
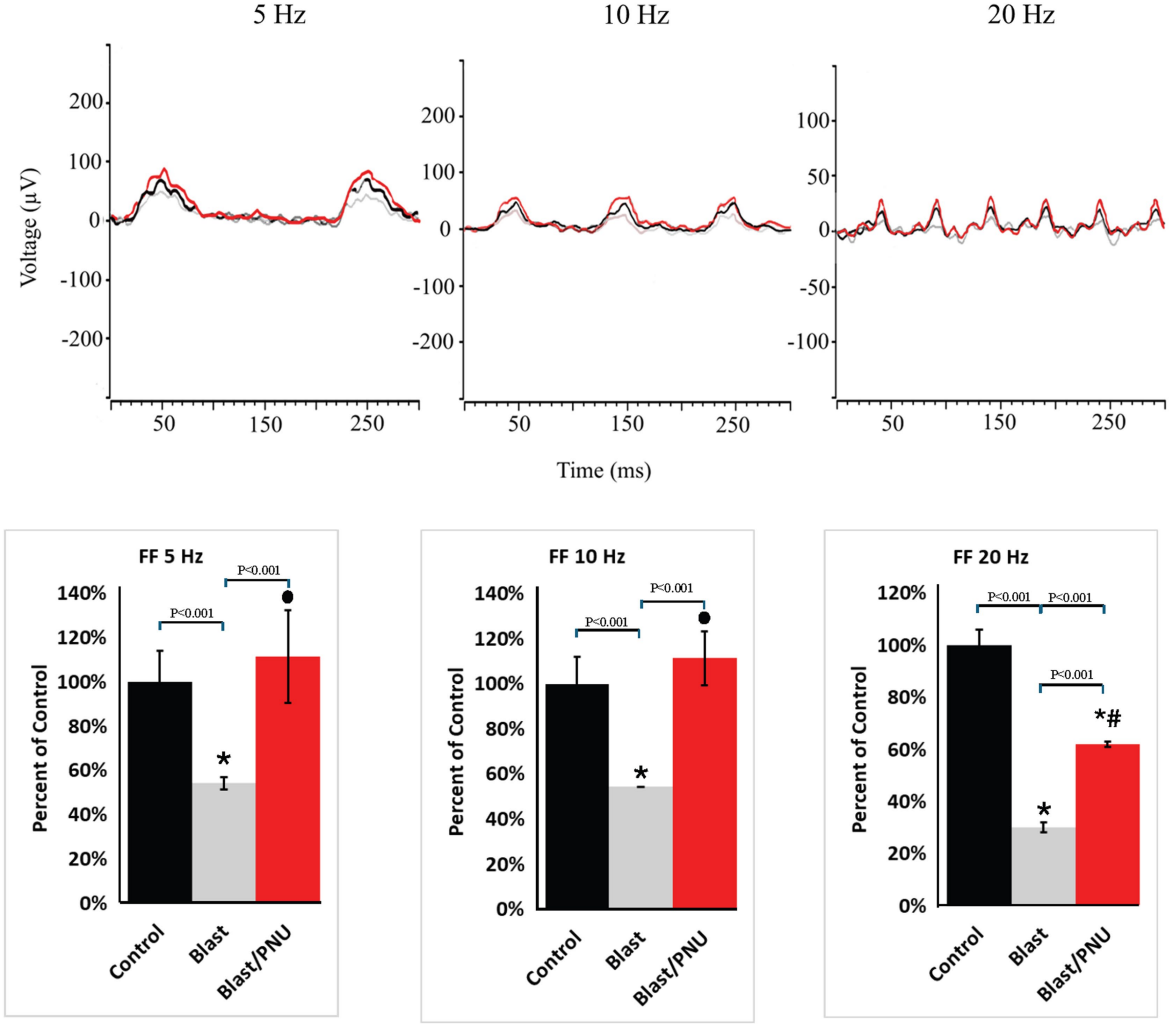
Recovery of flicker frequency in blast-exposed mice after treatment with PNU-282987. The superimposed ERG traces obtained from the same animal contain traces from control (black), blast only (gray), and blast with PNU-282987 (red) treated mice when stimulated at a flicker frequency of 5, 10 or 20 Hz. Quantification and averages of these responses from 6 to 8 animals are shown in the bar graphs below. * = significant difference from control; # represents a significant difference from control and from blast conditions, while the solid circle represents significant difference from blast only conditions.

The dark-adapted flicker frequency series was used to evaluate the ON- and OFF-cone-dependent pathways of the retina. Figure 7 shows examples of superimposed flicker frequencies at 5 Hz, 10 Hz, and 20 Hz obtained from a single animal under control conditions (black trace), after blast exposure (gray trace), and after blast exposure followed by the delayed PNU-282987 treatment (red trace), along with the corresponding quantification. We found that the amplitudes of the 5 Hz and 10 Hz responses, which represent the ON-cone response, were significantly decreased after blast exposure. Specifically, the 5 Hz amplitude decreased by an average of 45.7% ± 2.8%, and the 10 Hz amplitude decreased by 45.7% ± 0.2% compared to uninjured control retinas. The 20 Hz response amplitude, which is indicative of the OFF-cone response, was also significantly impacted, decreasing by an average of 69.8% ± 1.8%. Following treatment with PNU-282987, the 5 Hz and 10 Hz amplitudes were statistically similar to the amplitude recorded from the control untreated group. The 5 Hz response averaged 111.4% ± 21% of control recordings while the 10 Hz response averaged 111.4% ± 12% of the control recording. The 20 Hz response also showed significant recovery from the blast response, although it did not return to baseline levels. The average 20 Hz amplitude recovered to 62% ± 1% of its original response. No significant change in latencies were observed across the flicker frequency series.

### Functional recovery evaluated by ERG recordings: photopic negative response

**Figure 8:**
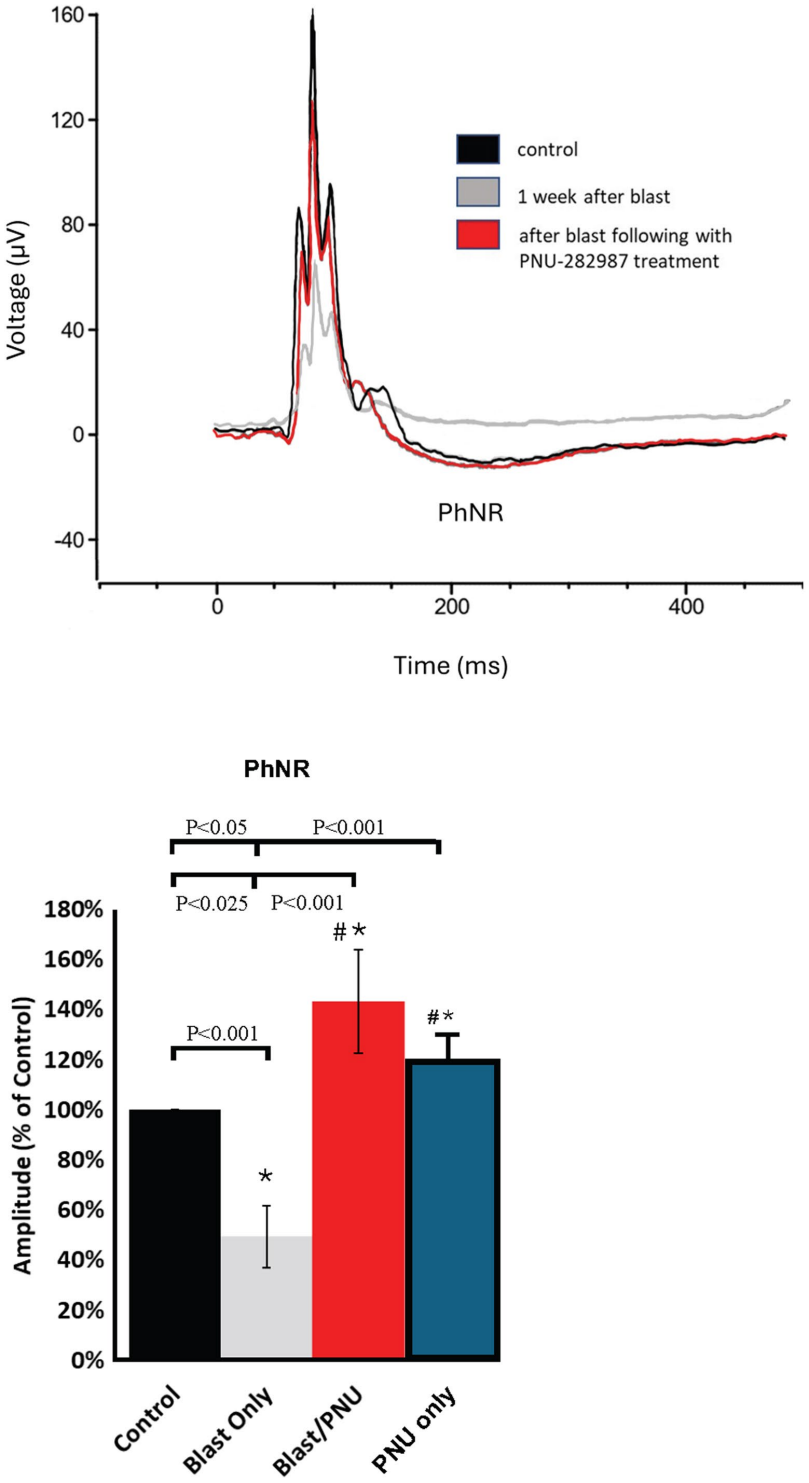
Recovery of PhnR amplitudes after treatment with PNU-282987 in blast exposed mice. The PhnR traces demonstrate the effects of blast exposure and PNU-282987 treatment. The superimposed PhnR traces contain traces from the same animal under control untreated conditions (black), after blast (gray), and after blast and PNU-282987 treatment (red). Quantification of average traces shown below (*N* = 6–8 for each bar graph shown). In summarized bar graphs, the blue bar represents a cohort of animals that were only treated with PNU-282987. * represents significant difference from control. # represents significant difference from control and from blast conditions. Error bar = SEM.

Analysis of the light-adapted PhNR can be used to assess RGC function as well as the parvocellular color pathway, giving an insight to study the impact of neuropathy and subsequent treatment on the innermost retinal layer (Ventura and Porciatti, 2006; Prencipe et al., 2020). In Figure 8, PhNR recordings from the same adult animal revealed that PhNR amplitudes significantly decreased after a single blast but demonstrated recovery after PNU-282987 treatment began 1 month following blast trauma. The bar graphs in Figure 8 quantify the average of each response. The PhNR negative amplitude significantly decreased by an average of 50.64% ± 12.2 following blast trauma when compared to control retinas. However, after PNU-282987 treatment, amplitudes significantly increased to 143.24% ± 20.6 compared to control conditions, representing a significant increase beyond the control response. In an additional study, ERG recordings were obtained from a cohort of animals that were only treated with PNU-282987. These results are summarized for the PhNR in the blue bar graph shown in Figure 8. PNU-282987 treatment by itself, significantly increased the amplitude of the PhNR corresponding with the increase of RGCs reported in Figure 5. No latency differences between controls, after blast, or after blast with PNU-282987 treatment were observed in the PhNR recordings.

### Functional recovery evaluated by ERG recordings: pattern electroretinogram

**Figure 9:**
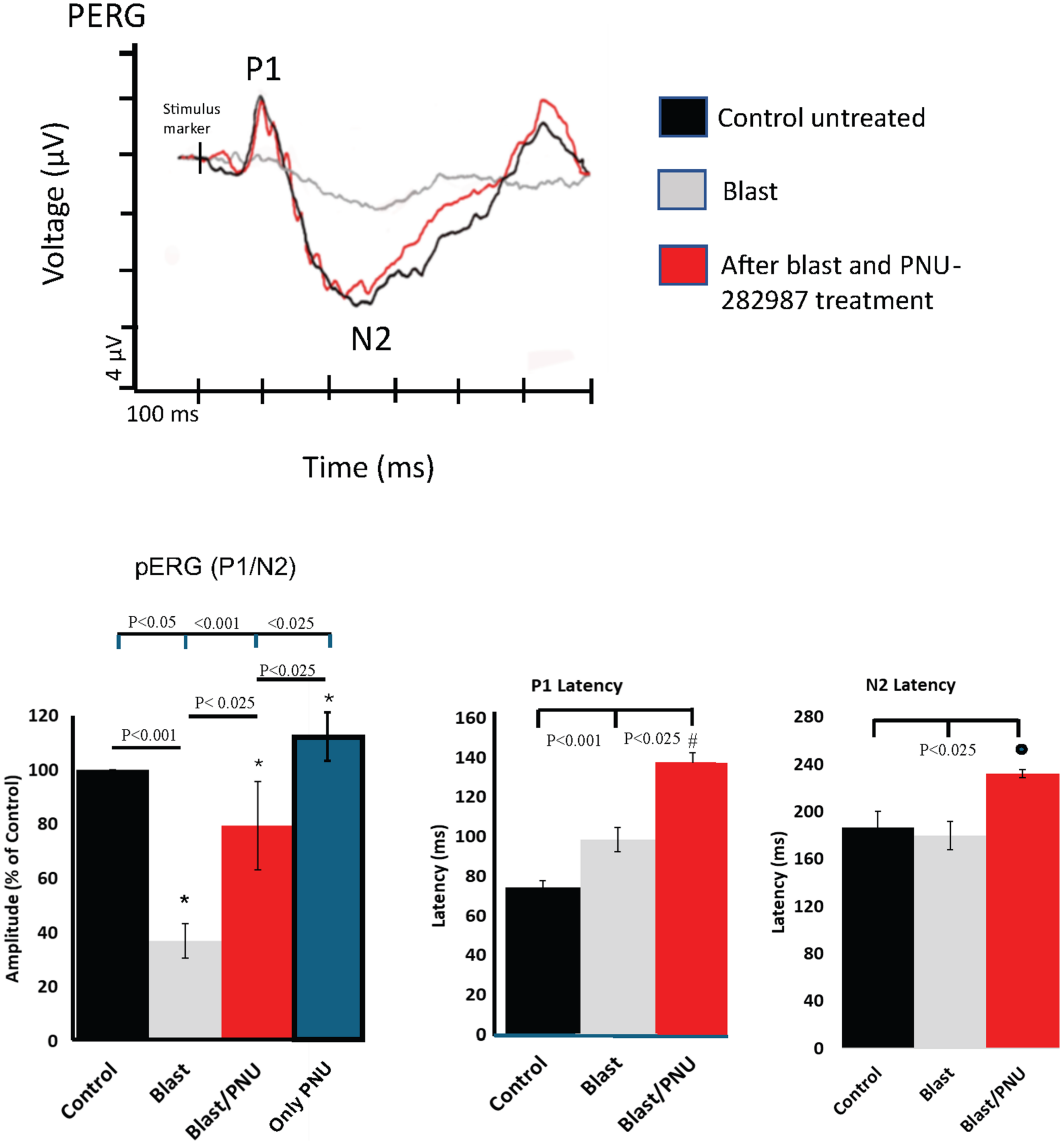
Recovery of pERG amplitudes after treatment with PNU-282987 in blast exposed mice. The pERG traces demonstrate the effects of blast exposure and PNU-282987 treatment. The superimposed pERG traces were obtained from the same animals and contain traces from control (black), blast (gray), and blast/PNU (red) treated mice. Quantification of average traces shown below in bar graphs for amplitudes of pERG P1/N2 and for latencies of P1 and N2. The blue bar graph for pERGs represents average results obtained when only PNU-282987 was added (N = 8 for each condition). *, # and the solid circle represents significant difference from all other conditions. Error bar = SEM.

Visual acuity and RGC function can also be assessed via the dark-adapted pERG. The pattern stimuli from the Celeris Diagnosys system includes alternating black and white checkerboard stimuli, which analyzes the RGC’s inherent contrast sensitivity and fine-detail capabilities (Fernández et al., 2024). As seen in the superimposed pERG traces shown in Figure 9, which were obtained from the same adult mouse, the P1/N2 amplitude of the recorded pERG significantly decreased following blast exposure (gray trace). Application of PNU-282987 resulted in a significant recovery of the P1/N2 pERG response to near control levels (red trace). The left bar graphs in Figure 9 quantify the average response under control conditions, after blast and after blast with the delayed PNU-282987 treatment. After blast, the average pERG P1N2 amplitude decreased by 55.22% ± 13.2. With PNU-282987 treatment following the blast, amplitudes significantly recovered by 68.32% ± 22.6 from blast levels. In an additional study, ERG recordings were obtained from a cohort of animals that were only treated with PNU-282987. These results are summarized for pERGs in the blue bar graph shown in Figure 9. PNU-282987 treatment by itself, significantly increased the amplitude of the pERG corresponding with the increase of RGCs reported in Figure 5.

Pattern ERG recordings indicated significant changes in latency following blast injury and PNU-282987 treatment in P1 and N2 waveform components. P1 waveforms revealed a progressive increase in P1 latencies following blast injury, from a mean latency of 74.25 ± 3.2 ms under control conditions, to 98.62 ± 6.1 ms after blast, and to 137.83 ± 4.5 ms after blast followed by PNU-282987 treatment. For N2 responses, latencies for control recordings were found to be a mean of 186.75 ± 13.42 ms, stayed consistent following blast injury with a mean of 179.66 ± 12.2 ms and statistically increased to a mean of 232 ± 3.4 with blast followed by PNU-282987 application. These findings are demonstrated and quantified in the middle and right bar graphs shown in Figure 9.

## Discussion

The experiments described in this study provide novel evidence of PNU-282987 as a neurogenic agent in the adult mammalian retina following blast-induced ocular trauma. This study is the first to analyze functional and morphological changes induced by an α7 nAChR agonist under a delayed treatment paradigm in the retina and expands upon the work of Spitsbergen et al. (2023). In the Spitsbergen et al., paper (2023), PNU-282987 treatment began immediately after a blast was delivered to adult mice and it was not certain if neuroprotection or neurogenesis occurred. The 2023 study demonstrated that a single blast exposure of 35 psi induced significant damage to retinal cells in the three nuclear layers of the retina and that immediate treatment with PNU-282987 mitigated cell loss and caused some recovery of ERG function. In this current study, treatment of PNU-282987 was delayed until after cell loss due to blast trauma that had already occurred, so that any mitotically labeled BrdU positive RGC or photoreceptor indicated a neurogenic effect instead of a neuroprotective effect.

Our results demonstrate that even with delayed treatment following traumatic injury, PNU-282987 is highly effective in promoting neurogenesis of retinal cells in an adult mammalian model. The presence of BrdU-positive cells across all retinal layers is a direct indicator of cellular proliferation, supporting the notion of robust neurogenesis taking place post-treatment. Specifically, co-labeling of BrdU with recoverin confirms the generation of new photoreceptors, and co-labeling of BrdU with Thy 1.2 provided evidence of new retinal ganglion cells after PNU-282987 treatment. These events were not observed in control, untreated retinas, reinforcing the hypothesis that the neurogenic activity in the retinas of adult mice were a direct result of PNU-282987 treatment. This finding is noteworthy, as the retina normally has a very limited regenerative capacity in adult mammals. Previous literature has provided examples of regeneration of new neurons in mammals, but the regeneration effect is minimal (Elsaeidi et al., 2018), unlike the robust neurogenesis that PNU-282987 induces. Inflammation following injury has also been shown to negatively impact adult mammalian neurogenesis (Monje et al., 2003; Ekdahl et al., 2003; Zhou et al., 2022; Bludau et al., 2024). This may have influenced the findings of Spitsbergen et al. (2023), in which PNU-282987 treatment was initiated immediately after blast exposure—potentially allowing inflammatory responses to counteract the drug’s neurogenic effects. In contrast, in the present study, treatment commenced 1 month post-injury, a timeframe in which inflammatory activity is likely significantly reduced (Saraiva et al., 2019), thereby enhancing the observed neurogenic response to PNU-282987.

Interestingly, latency changes were only observed during pERG recordings. There was no latency changes recorded from the a-wave, b-wave, or oscillatory potentials in simple flash ERGs under any condition, nor were there any significant latency changes recorded from flicker frequencies or PhNRs in any condition. The reason for this may lie in the stimulus type that separates these recordings. Pattern electroretinograms use a pattern reversing stimulus (Asanad and Karanjia, 2023) to intercept the visual acuity and contrast detection abilities of the retinal ganglion cells (Kim et al., 2021; Shamsi et al., 2022). This stimulus is more complex than the stimuli used for the other recordings such as the simple flash. The constant and rapid change in the pERG stimulus may introduce an inherent delay in visual processing time, which accounts for the observed latency changes. An alternative explanation is that the observed changes are too subtle to be detected within the short, eight-week timeframe of our study. Unlike glaucoma and other progressive retinal diseases where measurable changes in latency appear over many years, the blast injury we are examining is an acute event. In a different experiment from this lab where glaucoma was induced in a short period of time, a similar phenomenon was observed (Luzadre et al., 2025). mRNA sequencing results demonstrate that PNU-282987 induces a bimodal signaling event, where earlier activation is associated with an increased inflammatory response in the retina, followed by an inhibition of gliosis or scarring processes and decrease in immune response (Webster et al., 2022). This initial inflammatory process may be critical in the means by which PNU-282987 achieves neurogenesis and may also affect specific latency measurements before others.

Other laboratories have similarly explored activation of neurogenic pathways in the adult mammalian retina. Because MG exhibit reduced chromatin accessibility (VandenBosch et al., 2020), identifying molecular and extrinsic inhibitors of their regenerative capacity remains crucial. Several studies have shown that reprogramming MG into functional neurons can be achieved through delivery of proneural transcription factors, which drive formation of ganglion-like cells in adult mice (Pavlou et al., 2025). Similarly, genetic overexpression of these proneural transcription factors, such as Ascl1, has demonstrated enhanced MG-derived neurogenesis (Todd et al., 2021, 2022) and histone deacetylase inhabitation has been shown to increase chromatin accessibility and promote Ascl1-mediated regeneration following injury (Jorstad et al., 2017). Alternative strategies, such as differentiation of embryonic stem cells into photoreceptors for transplantation (Lamba et al., 2009; Gonzalez-Cordero et al., 2013) or blockade of inhibitory pathways including sFRP2 and BMP to stimulate endogenous progenitor proliferation (Balenci et al., 2013; Grisé et al., 2021), further support the potential for induced renewal in adult retinas. Nonetheless, despite advances in genetic and molecular interventions (Patel et al., 2017; Wohl et al., 2019), factors such as reactive gliosis (Dyer and Cepko, 2000; Vecino et al., 2016) and restrictive retinal microenvironment continue to limit full neuronal integration and functional recovery (Goldman, 2014; Miltner and La Torre, 2019). PNU-282987 itself is actively being explored as a potential therapeutic agent in a variety of other disorders and diseases (Duris et al., 2011; Zhou et al., 2017; Wang et al., 2020; Jiang et al., 2021; Linn, 2023; Sueyoshi et al., 2025), that are involved with the central nervous system (CNS). As the retina is an outpocketing of the CNS (London et al., 2013), the implications behind the PNU-282987-induced regeneration described in this research are profoundly relevant, as it may lend itself to further insight of treatments for many neurodegenerative disorders.

In this study, evidence was also provided to support the hypothesis that PNU-282987 restores electrophysiological function following blast trauma in adult mice. The functional recovery, as observed through ERG recordings, supports this hypothesis. For all ERG recordings, PNU-282987 treatment restored function to levels not significantly different from control after blast trauma, even with this delayed treatment paradigm. This finding strongly correlates with the formation of new neurons. In Spitsbergen et al. (2023), it was demonstrated that immediate treatment with PNU-282987 resulted in functional recovery that returned to near baseline levels, but it was not clear if PNU-282987 induced neuroprotection or neurogenesis. In our study, when experimental group populations were analyzed and normalized, blast exposure alone resulted in significantly reduced amplitudes compared to controls. In contrast, mice receiving both blast exposure and PNU-282987 treatment exhibited robust recovery, with amplitudes 8-weeks post injury not significantly different from control recordings. In rare instances where amplitudes exceeded 100% and were statistically significant, this may reflect increased number of cells, potential for integration into the retinal circuitry, and signal transmission to the occipital lobe. However, the mechanism of any response over 100% has not been characterized. Additionally, ERGs can only record the response to stimuli from the retina. Other recording techniques, including visual evoked potentials (VEPs), can lend insight into the functional effects of PNU-282987 in the brain, as VEPs record activity from the visual cortex (Creel, 2019). This lab has investigated previously whether the new neurons generated from PNU-282987 extend axons into the brain to form new synapses, further characterizing this phenomenon. In one study, lipophilic NeuroVue dye paper to label RGC axons was applied to the optic nerve of transgenic mice containing tamoxifen inducible TdTomato MG and demonstrated that new RGCs extend axons into the optic nerve following treatment with PNU-282987 (Spitsbergen et al., 2023). If we can elucidate the steps in which these neurons become functional after their generation, it could provide crucial insight into therapies for other neurodegenerative diseases and traumas.

The regenerative mechanism induced by PNU-282987 in adult mice remains under investigation. Prior studies from this lab showed that PNU-282987 stimulates MG to generate retinal neurons (Webster et al., 2017, 2019; Stanchfield et al., 2020; Webster et al., 2021, 2022). BrdU labeling revealed mitotically active cells across all retinal layers. However, a limitation of this approach is that the BrdU immunohistochemical protocol requires incubation of retinal tissue in 1 M hydrochloric acid (HCl). This step is essential for denaturing double-stranded DNA, allowing BrdU incorporation and subsequent antibody binding. Consequently, partial disruption of retinal integrity can occur, resulting in reduced cellular definition under confocal microscopy. To mitigate this, other studies from our laboratory have employed transgenic TdTomato mice, in which Müller glia and their progeny are fluorescently labeled, enabling lineage tracing of mitotic activity without compromising tissue structure. Complementary staining for MG (Vimentin, Sox9) and proliferative markers (Nestin, PCNA) confirmed BrdU localization to the ONL, INL, and GCL, with some co-labeling in MG (Webster et al., 2017, 2022). These findings support that PNU-282987 drives MG cell-cycle re-entry (Webster et al., 2019, 2021) and dedifferentiation into MDPCs, which subsequently can generate new retinal neurons. However, MG lack α7 nAChRs (Hendrickson et al., 2013; Paulo et al., 2009; Webster et al., 2019), and as a result the agonist likely acts indirectly through activation of α7 nAChRs on RPE cells. Previous studies determined that when MG cells were co-cultured with retinal pigment epithelial (RPE) cells treated with the α7 nAChR agonist in transwell experiments, upregulation of neurogenic genes occurred in MG after the RPE supernatant was injected into the vitreal chamber of adult wild type mice (Stanchfield et al., 2020). The present findings are consistent with this mechanism, as significant BrdU incorporation and co-labeling with photoreceptor and RGC markers reflect mitotic activation within a normally quiescent retina. Future studies aimed at identifying the ligand(s) released from the RPE that trigger MG reprogramming, through transcriptomic, proteomic, HPLC, and mass spectrometry analysis, will be critical to defining how PNU-282987 promotes retinal neurogenesis in adult mammals.

Neurogenic agents like PNU-282987 present a promising path toward more effective and lasting medical treatments. The findings of this study, alongside previous work, provide a strong basis for further research into stimulating new neuronal growth in adult mammals. If evidence of the underlying mechanisms for this neurogenic effect is understood, it could eventually lead to treatments for other types of nerve damage and neurodegenerative diseases like Alzheimer’s and Parkinson’s disease. While treating neurodegeneration and neural injuries remains challenging, agents, such as PNU-282987, offer an avenue for significantly improving the quality of life for individuals affected by neurodegenerative diseases. For those impacted by blast-induced ocular trauma, a devastating consequence of modern conflict, the implications of this research extend far beyond the battlefield. By providing insight into the mechanisms behind unlocking neurogenesis in adult mammals, PNU-282987 can become a precursor for treatments used in active combat, for veterans, and civilians impacted by war. In a world where ocular trauma is a leading cause of blindness and neurodegenerative diseases are increasing, this agonist offers a shift from symptom management to an active approach of neural repair.

## Data Availability

The raw data supporting the conclusions of this article will be made available by the authors, without undue reservation.

## Glossary

PNU-282987: N-[(3’R)-1′-azabicyclo [2.2.2] oct-3′-yl]-4-chlorobenzamide hydrochloride
BrdU: bromodeoxyuridine
ERG: electroretinogram
MG: Müller Glia
MAPK: mitogen-activated protein kinase
FGF-2: fibroblast growth factor 2
hERG: human ether-a-go-go related gene
RP: retinitis pigmentosa
RGCs: retinal ganglion cells
mRNA: message RNA
RPE: retinal pigment epithelium
nAChRs: nicotinic acetylchloline receptors
psi: pound-force per square inch
FFS: flicker frequency series
PhNR: phototopic negative response
pERG: pattern electroretinogram
PBS: phosphate buffer
DMSO: dimethyl sulfoxide
HPLC: high pressure liquid chromatography
C0_2_: carbon dioxide
PFA: paraformaldehyde
IHC: immunocytochemistry
DAPI: 4′,6-diamidine-2′-phenylindole dihydrochloride
ONL: outer nuclear layer
INL: inner nuclear layer
GCL: ganglion cell layer
KAX: ketamine/acepromazine/xylazine
IP: intraperitoneal
LED: light emitting diode
OP: oscillatory potential
